# The Developing Mental Number Line: Does Its Directionality Relate to 5- to 7-Year-Old Children’s Mathematical Abilities?

**DOI:** 10.3389/fpsyg.2018.01142

**Published:** 2018-07-06

**Authors:** Lauren S. Aulet, Stella F. Lourenco

**Affiliations:** Department of Psychology, Emory University, Atlanta, GA, United States

**Keywords:** spatial-numerical associations, mental number line, SNARC, mathematical ability, development

## Abstract

Spatial representations of number, such as a left-to-right oriented mental number line, are well documented in Western culture. Yet, the functional significance of such a representation remains unclear. To test the prominent hypothesis that a mental number line may support mathematical development, we examined the relation between spatial-numerical associations (SNAs) and math proficiency in 5- to 7-year-old children. We found evidence of SNAs with two tasks: a non-symbolic magnitude comparison task, and a symbolic “Where was the number?” (WTN) task. Further, we found a significant correlation between these two tasks, demonstrating convergent validity of the directional mental number line across numerical format. Although there were no significant correlations between children’s SNAs on the WTN task and math ability, children’s SNAs on the magnitude comparison task were negatively correlated with their performance on a measure of cross-modal arithmetic, suggesting that children with a stronger left-to-right oriented mental number line were less competent at cross-modal arithmetic, an effect that held when controlling for age and a set of general cognitive abilities. Despite some evidence for a negative relation between SNAs and math ability in adulthood, we argue that the effect here may reflect task demands specific to the magnitude comparison task, not necessarily an impediment of the mental number line to math performance. We conclude with a discussion of the different properties that characterize a mental number line and how these different properties may relate to mathematical ability.

## Introduction

Interest in the spatial nature of numerical representations can be traced back as early as 1880 to Francis Galton’s work on “number forms.” In this work, Galton demonstrated that individuals visualized numbers in spatial format, albeit in an idiosyncratic manner across individuals (Galton, 1880). In subsequent, now seminal work, Dehaene et al. (1993) documented systematic associations, the so-called SNARC (spatial-numerical association of response codes) effect, among Western participants who made parity (odd/even) judgments to Arabic numerals using left and right response keys. In this study, participants responded faster to smaller numbers when using the left key and responded faster to larger numbers when using the right key, providing evidence of a left-to-right spatial representation of number (see also Zorzi et al., 2002; Fischer et al., 2003; Wood et al., 2008), often referred to as the mental number line^1^. In the 25 years since the publication of this work, there have been ongoing efforts to understand the ontogenetic and phylogenetic origins of such a number line (for alternative perspectives, Gevers et al., 2003; Abrahamse et al., 2016). Because this initial work dealt primarily with symbolic numerical stimuli (Dehaene et al., 1993), and because it has since been shown that there is cross-cultural variation in the direction of these effects (Zebian, 2005; Shaki et al., 2009), it was hypothesized that the mental number line arose from experience with linguistic conventions (i.e., reading and writing). Although cultural experience certainly modulates the directionality of one’s mental number line (Shaki et al., 2009; McCrink et al., 2014), recent research using non-symbolic stimuli demonstrates directional effects in non-human animals (Drucker and Brannon, 2014; Rugani et al., 2015) and preliterate children (Patro and Haman, 2012; de Hevia et al., 2014), though the specific orientation of these directional effects may vary (Cooperrider et al., 2017; Gazes et al., 2017).

Yet, there are open questions regarding both the developmental trajectory and functional significance of a mental number line. To address such questions, it is important to acknowledge that the mental number line is a heterogeneous phenomenon. There are both directional and non-directional properties of the mental number line (for review, see Cipora et al., 2015). The directional property of the mental number line refers specifically to the orientation of the mapping between numbers and space. For example, Westerners orient numbers left-to-right, in contrast to non-Westerners who may orient numbers right-to-left (Shaki et al., 2009). Among the non-directional properties is the type of spatial scaling (see recent meta-analysis, Schneider et al., 2018). For example, there is a large literature on the extent to which the mental number line can be considered linear as opposed to compressive (e.g., logarithmic; Siegler and Opfer, 2003; Dehaene et al., 2008; Lourenco and Longo, 2009). This literature has provided evidence for a developmental shift from compressive to linear number representations (Siegler et al., 2009; Opfer et al., 2016) as well as for a relation between spatial scale and math development. In particular, children’s performance on a variety of math tasks has been found to be positively associated with the linearity of their representations (Siegler and Booth, 2004; Booth and Siegler, 2006; Sasanguie et al., 2013). However, compared to the non-directional properties of the mental number line, far less work has concerned the development and function of the directional nature of the mental number line, particularly the potential relation between directionality and math competence. Thus, the present study focused specifically on the development and function of SNAs as a way to ask whether directionality, like spatial scaling, affords any benefit to math development.

Assessing SNAs in children has proven difficult, as most tasks designed for adults utilize relatively advanced numerical judgments (i.e., parity), bimanual responses, and/or reaction time (RT) data, which may be unsuitable for children or pose interpretative challenges when testing developmental populations. To address these challenges, Opfer et al. (2010) implemented a search task in which they found that 4-year-olds were more accurate at locating an item in a series of horizontally arranged containers when the containers were numbered from left-to-right, as opposed to right-to-left (see also Opfer and Furlong, 2011). Similarly, they found that children counted a series of horizontally arranged items from left-to-right more often than from right-to-left. Though this work suggests that young children are sensitive to culturally specific counting practices (Shaki et al., 2012), it is less clear whether their performance was driven by access to a left-to-right mental number line.

Other researchers have devised tasks that minimize the demands imposed on children while also maintaining similarity to those used with adults in an effort to address questions about the developmental continuity of SNAs. For example, van Galen and Reitsma (2008) adopted magnitude, instead of parity, judgments (i.e., Is the presented Arabic numeral smaller or larger than 5?) and found evidence of SNAs in 7- to 9-year-old children, as has been shown in adults. In this study, children responded faster to smaller numbers when using the left key and responded faster to larger numbers when using the right key, consistent with a left-to-right mental number line. Patro and Haman (2012) further addressed the methodological challenges of testing children with a non-symbolic magnitude comparison task (i.e., a comparison of dot displays) and unimanual responses. In their study, 2- to 4-year-olds judged which of two simultaneously presented arrays contained more items by selecting the array with the larger numerosity. They found that children were significantly faster when the target array was on the right side of space than on the left. They also showed a similar, albeit weaker, effect when children selected the array that was smaller in numerosity, such that children were somewhat quicker when the target array was on the left. Taken together, these findings provide support for a directional mental number line that emerges early in development, when experience with reading and mathematics is minimal. However, even with some evidence for directional number representations beginning in infancy (Bulf et al., 2016), the reliability of these effects remains unknown. Moreover, and crucially, little is known about the potential functional significance of such number representations. In particular, we can ask whether the directionality of the mental number line has any impact on one’s understanding of mathematical concepts and reasoning.

### Does the Directionality of the Mental Number Line Have Functional Significance?

One prominent hypothesis is that a mental number line functions to support mathematical development and understanding (Opfer et al., 2010; Fischer and Shaki, 2014). On this view, stronger SNAs would be accompanied by better math ability. Although evidence of SNAs in non-human animals would seem to argue against this perspective (since non-human animals never develop formal math skills), it remains possible that, at least in humans, mathematical reasoning recruits numerical representations shared by humans and non-human animals. This possibility is buttressed by research on the approximate number system (ANS; Feigenson et al., 2004), which has found that the ANS, a non-verbal system of number representation that humans, similarly, share with non-human animals (Cantlon and Brannon, 2006; Rugani et al., 2008; Agrillo et al., 2011) and that is operational early in human development (Xu and Spelke, 2000; Cordes and Brannon, 2008), predicts formal math abilities when assessed concurrently (Libertus et al., 2011; Bonny and Lourenco, 2013) and longitudinally (Halberda et al., 2008; Starr et al., 2013).

Yet, existing research on the relation between SNAs and mathematical ability conducted on adult participants does not provide strong evidence for a directional mental number line that benefits mathematical reasoning. In particular, one line of evidence suggests a negative relation, such that participants with math difficulties showed stronger SNAs than participants without math difficulties (Hoffmann et al., 2014a). Likewise, non-mathematicians (i.e., doctoral students in the humanities and social sciences) have been found to show stronger SNAs than mathematicians (i.e., doctoral students in mathematics; Cipora et al., 2016). Other studies, however, have reported no relation between SNAs and math competence in adulthood (Bull et al., 2013; Cipora and Nuerk, 2013), demonstrating inconsistency in the extant research with adults.

The challenge with using adult participants to test whether SNAs may be related to mathematical reasoning is that adults have a mature system of mathematics at their disposal. Although there is certainly variability in the math exposure adults experience, most adults in Western society have learned a variety of mathematical concepts and are capable of performing computations on numbers and variables. For these reasons, one might ask whether the spatial instantiation of mathematical concepts would be of greater utility earlier in development, when these concepts are initially acquired and remain difficult to grasp. Recent evidence suggests that early spatial skills such as mental rotation predict later math development (Lauer and Lourenco, 2016; Verdine et al., 2017), suggesting a potentially broader role for spatial representations in math learning. As highlighted above, however, SNAs have been difficult to assess in children, impeding the study of their potential utility at the critical early stages of math development. To date, only a few studies have assessed the relation between SNAs and math ability in childhood, and, as with adults, the findings have been inconsistent.

Hoffman et al. (2013) tested the relation between SNAs and math ability in 5-year-old children who completed color and magnitude judgments on Arabic numerals (similar to the task used by van Galen and Reitsma, 2008). Children also completed measures of numerical competence (e.g., verbal counting and digit writing). Although children showed evidence of an SNA when judging the color of Arabic numerals, extending the finding of van Galen and Reitsma (2008) to younger children, individual performance did not correlate with any measure of numerical competence. By contrast, when SNAs were indexed using the magnitude judgment task, there was a positive correlation between children’s SNAs and some measures of numerical competence. Importantly, however, the SNA effect, at the group-level, was not significant, raising questions about the reliability of children’s SNAs on the magnitude judgment task in this study and, thus, the reported links with numerical competence.

In other work, Bachot et al. (2005) examined a group of 16 children from ages 7 to 12 years with visuospatial deficits, some of whom also had dyscalculia, a mathematical learning disability. They found that children with visuospatial and mathematical deficits did not exhibit SNAs on a symbolic magnitude judgment task, whereas a control group of children, matched for age and gender, did. However, because children with visuospatial and math deficits were not differentiated in the analyses, it is unclear whether the lack of a significant SNA was driven by poor math ability or visuospatial deficits. More recently, Gibson and Maurer (2016) used a similar symbolic magnitude comparison task to assess SNAs in 6- to 8-year-old typically-developing children. They found that 6-year-olds, like the children in the study of Hoffman et al. (2013), did not show an SNA on this task. However, in older children (7- and 8-year-olds), the SNA effect was significant, but this effect did not relate to math performance, as assessed by a standardized measure of symbolic math ability (for similar results, see Schneider et al., 2009). In a study of 8- to 11-year-olds (*n* = 55), Georges et al. (2017b) assessed children’s SNAs for symbolic numerals with a parity judgment task. They observed a positive relation between children’s SNAs and performance on the arithmetic, but not visuospatial, subtest of a standardized, speeded math exam, such that children with stronger SNAs exhibited better arithmetic skill. However, this relation was only observed in younger children (8–10 years old), in contrast with Gibson and Maurer (2016), who observed no relation between SNAs and performance on a standardized assessment of symbolic math in children of similar age.

Taken together, the extant data from studies with children suggest that a mental number line, with consistent directionality, may be present as young as preschool age (for work with infants, see Bulf et al., 2016). However, the evidence in support of a relation between an early emerging directional mental number line and mathematical development is mixed, with several open questions following from the existing findings. For example, it remains unknown how associations between symbolic and non-symbolic representations of number affect the link between SNAs and math competence, given that a directional mental number line with symbolic numerals may not be present until 7 years of age. As in studies with adults, it is also unclear whether the relation between SNAs and math competence in children depends on the type of math ability assessed such as whether arithmetic computations are performed exactly or approximately.

### Present Study

In the present study, we assessed children’s performance on two SNA tasks, as well as on multiple measures of early mathematical competence. Children were between 5 and 7 years of age, an age range in which formal math instruction has only recently begun and, thus, when even basic mathematical concepts and operations may not yet be mastered. The two measures of SNAs assessed the directionality of children’s number representations (mental number line) using different judgments and either symbolic or non-symbolic stimuli. One SNA task was a non-symbolic version of the magnitude comparison task (Patro and Haman, 2012), and the other SNA task was a novel “Where was the number?” (WTN) task with Arabic numerals (adapted from Aulet et al., 2017). In this task, children simply viewed a number on a screen, memorized its location, and, after a short delay, placed the number back in its original location. As noted previously, existing research using Arabic numerals in a magnitude comparison task has not provided evidence of SNAs until approximately 7 years of age. However, the WTN task required no explicit judgment of magnitude, but rather, only memory for the location of a number that had appeared in a random location on screen, which we reasoned might allow for earlier detection of the directionality of the mental number line with symbolic stimuli. Moreover, the differences in stimuli and task requirements across these two tasks provided a strong test of construct validity. In other words, if a stable, directional mental number line underlies performance on both tasks, then children’s performance on the two tasks should be correlated.

We also examined the relation between children’s performance on the SNA tasks and their mathematical ability (see **Table 1** for all tasks used in the present study). Because math is not a monolithic concept and, crucially, because the link between SNAs and math ability may depend on the type of math that is assessed, we included multiple measures of early math ability. One possibility is that the understanding of the abstract nature of number would benefit from a grounding in space (for review, see Lourenco et al., 2018). Indeed, the directionality of the mental number line could support the understanding of number as an abstract concept with ordinal structure (Cipora et al., 2015). Another possibility is that this directionality could provide support for the enactment of arithmetic operations, as suggested by the spatial-directional biases associated with addition and subtraction, known as “operational momentum” (McCrink et al., 2007; Pinhas and Fischer, 2008; see also: Klein et al., 2014; Holmes et al., 2016). In particular, it has been suggested that addition and subtraction elicit rightward and leftward movement, respectively, along the mental number line. The mental number line could provide a concrete method for instantiating the arithmetic operations by distinguishing addition and subtraction in terms of directional movement and perhaps by supporting implementation of the computation (Booth and Siegler, 2008; Siegler, 2016). In the present study, we tested children on tasks designed to target these areas of emerging math competency. In particular, children completed a task that required coordination of numerical information across modalities (vision and audition), as an assessment of children’s abstract number representations. Specifically, this task served as a test of abstractness since children must “abstract” across the perceptual information to achieve a common number representation across format. Children also completed two measures of symbolic arithmetic (one approximate and one exact) that assessed competence with arithmetic computation on numerals.

**Table 1:** Description of the tasks used in the present study and the constructs they were designed to assess.

Task	Construct	Number format	Calculation type
‘Where was the number?’ (WTN)	Spatial-Numerical Associations	Symbolic	N/A
Magnitude Comparison	Spatial-Numerical Associations	Non-symbolic	N/A
Approximate Cross-modal Arithmetic (ACA)	Math Ability	Non-symbolic	Approximate
Approximate Symbolic Arithmetic (ASA)	Math Ability	Symbolic	Approximate
WJ – Calculation^∗^	Math Ability	Symbolic	Exact
WJ – Auditory Working Memory^∗^	Verbal Working Memory	N/A	N/A
WJ – Picture Vocabulary^∗^	Verbal Proficiency	N/A	N/A
K-ABC – Spatial Memory^∗^	Spatial Short-Term Memory	N/A	N/A


*For numerical tasks, we further specify the number format used and the calculation type assessed. ^∗^Standardized tasks.*

Furthermore, we assessed the internal consistency of all SNA and math tasks in the present study to ensure that any observed relations (or lack thereof) between SNAs and math ability could not be attributed to poor task reliability. Although assessment of reliability is especially critical when utilizing an individual differences approach, previous studies on the relation between SNAs and math ability have infrequently reported the reliability of measures. Finally, to assess the specificity of the link between children’s SNAs and their developing math abilities, we included several tasks to control for general cognitive functioning. These tasks assessed verbal proficiency (WJ–Picture Vocabulary subtest; Woodcock et al., 2001a), verbal working memory (WJ–Auditory Working Memory subtest; Woodcock et al., 2001b), and spatial short-term memory (Spatial Memory subtest of the Kaufman-Assessment Battery for Children (K-ABC); Kaufman and Kaufman, 1983). Previous studies reporting a relation between SNAs and math ability have not controlled for general cognitive functioning, leaving open the possibility that other abilities shared by a directional mental number line and math tasks could account for the reported relation. Here we directly addressed this possibility.

## Materials and Methods

### Participants

Sixty-six children (28 female) between the ages of 5 and 7 years of age (*M* = 74.65 months, *SD* = 9.62 months) from the greater Atlanta area participated in this study. One child was excluded from the analyses for failing to complete multiple tasks. Caregivers provided written informed consent on behalf of their children. All children received stickers throughout the session to maintain motivation, as well as a small gift at the end of the session for participating in the study. Experimental procedures were approved by the local ethics committee.

### Tasks and Procedure

#### “Where Was The Number?” Task

In the “WTN” task (adapted from Aulet et al., 2017), children viewed an Arabic numeral (1–9) presented in black font within a rectangle [white fill with black outline; 915 × 495 pixels (24.29 cm× 13.10 cm)]. At the start of each trial, a number appeared at a random location within the rectangle (the “whiteboard”). Children were instructed to press a virtual button located at the bottom of the screen (“START”) once they felt they had sufficiently memorized the location of the number. When the start button was pressed, the number disappeared and an image of a dry-erase marker appeared, presented centrally. This was done to ensure that children did not visually fixate on the original location of the number and that all children initiated their responses from the same starting location. Next, children tapped the image of the dry-erase marker, which then disappeared. Children then made their responses by tapping the location on the whiteboard where they believed the number previously appeared. The number appeared at the tapped location. Adjustments to responses could be implemented by tapping and dragging the number to a new location. When satisfied with the placement of the number, children pressed a virtual button located at the bottom of the screen (“Done!”) to confirm their response and they proceeded immediately to the next trial.

Presentation of numbers and duration of response window were untimed. Children completed 72 trials in total (each number presented eight times each). To ensure that children remained attentive throughout the task, trials were split into four blocks, each consisting of 18 trials (each number presented twice; random order).

#### Magnitude Comparison Task

Following Patro and Haman (2012), children completed “more” and “less” conditions of our magnitude comparison task (order counterbalanced across children). In the more condition, children were asked to judge which of two dot arrays was larger in numerosity. In the less condition, children were asked to judge which of two arrays was smaller in numerosity. Following Gebuis and Reynvoet (2011), non-numerical properties in these arrays, such as element size and convex hull, were varied across trials to ensure no systematic relation between these properties and numerosity. Numerical arrays (13.72 cm × 13.72 cm) were arranged horizontally on screen, each below an image of a Star Wars character (BB-8 and R2D2).

In each condition, children completed three practice trials in which they were given corrective feedback. In the practice trials, the two arrays differed in numerosity by a 1:2 ratio (i.e., arrays of 4 vs. 8, 5 vs. 10, and 8 vs. 16). In the test trials, the two arrays differed in numerosity by a 4:5 ratio (i.e., arrays of 4 vs. 5, 8 vs. 10, 12 vs. 15, and 16 vs. 20). Children completed 16 test trials (each ratio presented four times) in each condition, for a total of 32 test trials. On half of the trials, arrays were presented in the congruent position, with the numerically smaller array presented on the left and the numerically larger array presented on the right. On the other half of the trials, arrays were presented in the incongruent position, with the numerically smaller array presented on the right and the numerically larger array presented on the left. Following previous research (Mazzocco et al., 2011), arrays were visible for 1,200 ms before being occluded. Arrays remained occluded until children responded and then proceeded to the next trial (1,500 ms ISI). All responses were made on a touchscreen.

#### Approximate Cross-Modal Arithmetic (ACA) Task

In the ACA task (adapted from Barth et al., 2008), we measured the extent to which children’s representations of number were modality independent by testing their ability to perform addition and subtraction across displays of dots and sequences of tones. At the beginning of each condition, children completed a familiarization phase as well as two practice trials. In the familiarization phase, children were shown an example animation in which the appearance (addition condition) or disappearance (subtraction condition) of blue dots, one-by-one, was paired with a tone. After this animation, a new array of blue dots was displayed (dots presented simultaneously) and was then occluded by a matching blue occluder. They were told that if they listened carefully, they would hear more blue dots “appear” or “disappear,” at which time they heard a sequence of tones. The experimenter then asked the child whether there would be more or less dots behind the occluder than before. If children answered correctly in these demonstrations (“more” for addition and “less” for subtraction), then the experimenter proceeded to the practice trials. If children answered incorrectly, then the experimenter repeated the previous animations.

Children were given two practice trials in which an array of blue dots (19.30 cm × 13.72 cm) was displayed on the left side of the screen (dots presented simultaneously) and was then occluded. After occlusion, children heard a sequence of tones, representing the appearance/disappearance of blue dots. While the blue occluder remained on screen, an array of red dots appeared (19.30 cm × 13.72 cm) on the right side of the screen that was then covered by a matching red occluder (arrays and occluders were matched for luminance). Children were asked whether there were more dots behind the blue or red occluder. After their response, the experimenter removed the occluders to reveal both arrays, providing children corrective feedback. In both practice trials, blue and red dots differed by a 1:2 ratio (one trial with more blue dots and one trial with more red dots). Following a response, the experimenter advanced to the next trial.

After the practice trials, children completed 12 test trials (randomly ordered). In these trials, blue and red dots differed by one of three ratios: 4:5, 4:6, or 4:7. Children completed four trials of each ratio (two trials in which the blue array was more numerous and two trials in which the red array was more numerous). As in Barth et al. (2008), element size was held constant on all trials of this task. Importantly, though, reliance on non-numerical cues was not likely to account for performance on this task, as success on the task required addition/subtraction of elements across vision and audition (in which the cues differed). The same tone (duration = 15 ms) was used in all tone sequences. This tone was repeated multiple times in each sequence, presented in an irregular rhythm. In the addition condition, final set sizes (dots plus tones), ranged from 16 to 54 (*M* = 35). In the subtraction condition, final set sizes (dots minus tones) ranged from 7 to 30 (*M* = 16). The duration of the sequence of tones ranged from 1.70 to 3.70 s. Although these durations were likely too fast to allow for consistent counting, children were told at the start of the task not to count the individual items and any child who displayed evidence of counting was immediately instructed not to do so. This procedure was used to ensure that all children added or subtracted the sequences using the same approximation strategy.

At the start of each test trial, an array of blue dots (against a solid black background) was displayed on the left side of the screen for 3 s. Then, the blue array was occluded and remained occluded for 6 s while the sequence of tones played. Following this presentation, an array of red dots was displayed on the right side of the screen for 3 s and was then occluded. In all trials of the ACA task, the first display was presented on the left side of the screen and the second display was presented on the right side of the screen. Items were always added to, or subtracted from, the first display^2^. Children were only permitted to respond which array was more numerous once both arrays were occluded. Responses were made using the touchscreen. Immediately after children made their response, the experimenter pressed a key to proceed to the next trial. After children completed the addition condition, the same procedure was completed for the subtraction condition. All children completed the addition condition prior to the subtraction condition. We fixed the trial order in this way because previous research has found that subtraction can be more difficult than addition (Barth et al., 2008) and it has been shown that difficult trials negatively impact performance on subsequent trials (Odic et al., 2014). Thus, to avoid negative carryover effects, addition trials were administered prior to subtraction trials.

#### Approximate Symbolic Arithmetic (ASA) Task

We assessed children’s ability to engage in ASA by requiring them to solve addition and subtraction problems without engaging in exact computation (adapted from Gilmore et al., 2007). In this task, problems were presented verbally along with visual displays containing Arabic numerals. An example problem was: “Sarah has 20 candies in her bag, and then she gets 25 more. John has 30 candies. Which one of them has more candies?” On these problems, the visual displays consisted of cartoon children with accompanying Arabic numerals. Like the ACA task, the first display (e.g., character) was presented on the left side of the screen and the second display was presented on the right side of the screen. Items were always added to, or subtracted from, the first display^2^. Following the reading of the quantities, the corresponding visual displays containing the Arabic numerals were occluded to discourage exact calculation. After the experimenter finished presenting the problem, children responded by pointing to, or naming, the character who they judged as having more candies. Children completed two conditions: addition and subtraction. Within each condition, children completed 12 trials. In the addition condition, final set sizes ranged from 12 to 58 (*M* = 30). In the subtraction condition, final set sizes ranged from 10 to 56 (*M* = 28). Within each trial, final set sizes differed by one of three ratios: 4:5, 4:6, or 4:7. Trials were randomly ordered and untimed. As with the ACA task, the order was fixed to prevent negative carryover effects (Odic et al., 2014), such that the addition condition was administered prior to the subtraction condition.

#### Exact Symbolic Arithmetic Task

Children completed the Calculation subtest of the Woodcock Johnson (WJ) Tests of Achievement (Woodcock et al., 2001a), a standardized assessment of exact symbolic arithmetic ability. Specifically, the WJ–Calculation test measures participants’ ability to perform exact computation using addition, subtraction, multiplication, and division with whole numerals. This test is untimed and administered in paper-and-pencil format following a standard protocol, such that testing is discontinued once six consecutive questions are answered incorrectly.

#### Control Tasks

Children completed two subtests from the WJ Tests of Achievement and WJ Tests of Cognitive Abilities (Woodcock et al., 2001a,b) that served as controls for general cognitive functioning: verbal proficiency (WJ–Picture Vocabulary) and verbal working memory (WJ–Auditory Working Memory). Children also completed the Spatial Memory subtest from the K-ABC (Kaufman and Kaufman, 1983) as an assessment of spatial short-term memory and to serve as another non-math control task. All control tasks were untimed (for procedural details corresponding to each task, see Kaufman and Kaufman, 1983; McGrew et al., 2007). All control tasks have acceptable reliability, as determined by a split-half procedure: WJ Picture Vocabulary, *r* = 0.81; WJ Auditory Working Memory, *r* = 0.96; K-ABC Spatial Memory, *r* = 0.80 (Kaufman and Kaufman, 1983; McGrew et al., 2007).

#### General Procedure

All computerized tasks were presented on a Hewlett Packard Compaq Elite 8300 23″ all-in-one desktop computer (resolution: 1920 × 1080 pixels). Children were tested individually by an experimenter. Children sat approximately 40 cm from the screen for all computerized tasks. For ease of administration, a fixed order was used such that tasks requiring similar materials were administered consecutively, with computerized tasks preceding paper-and-pencil tasks. Of the computerized tasks, all children first completed the magnitude comparison task, followed by the ACA and ASA tasks (counterbalanced order). Of the paper-and-pencil tasks, all children first completed the WJ–Calculation test. Then, children completed the three control tasks in a randomized order. Given concerns about children’s attentiveness across trials, children completed four separate blocks of the WTN task. These blocks were administered at fixed points throughout the session: at the start of the session, as the first task (block 1); after the magnitude comparison task (block 2); after the ACA and ASA tasks (block 3); and after the completion of all standardized tasks, as the last task (block 4). Following the completion of each task, children received a sticker.

## Results

### Preliminary Analyses

Preliminary analyses showed that scores on all tasks, except for the WTN task, were normally distributed, with skewness statistics within an acceptable range (±0.60; Tabachnick and Fidell, 2001). Scores on the WTN task were transformed (square root transformed) for the correlation analyses reported in the following sections; skewness on the WTN task was in an acceptable range following the transformation.

All tasks yielded acceptable reliabilities (*r*s > 0.52, Spearman–Brown corrected; see **Table 2**). Reliabilities were calculated for each task using a sample-with-replacement bootstrap technique following Anobile et al. (2016). For each child, the dependent variable (i.e., congruency score for the Magnitude Comparison task, slope for the WTN task, and accuracies for the ACA and ASA tasks) was calculated twice from a random sample of the data (half of the total number of trials for the respective task). This sampling procedure was trial blocked such that equal numbers of each trial type were included (e.g., an equal number of trials for each operation and ratio in the ACA and ASA tasks). We then computed the correlation between the two values, across subjects. This process was repeated 1,000 times and we calculated reliability as the mean correlation for each task.

**Table 2:** Partial correlations between SNA tasks and the different math tasks (dependent variable for each task in parentheses), controlling for age. Also included are descriptive statistics for each task.

	Task	1	2	3	4	5	*n*	*M*	*SD*	Reliability
1	WTN (slope)		0.012	0.316	0.100	0.747	58	1.07	4.06	0.71
2	Magnitude comparison (congruency score)	0.339^∗†^		0.021	0.770	0.153	64	0.594	2.23	0.52
3	ACA (proportion correct)	-0.148	0.313^∗^		0.356	0.690	56	0.659	0.114	0.72
4	ASA (proportion correct)	0.248	-0.042	0.138		0.055	53	0.755	0.148	0.86
5	WJ–Calculation (raw scores)	0.044	-0.182	0.055	0.267		54	6.71	4.21	0.93^∧^


*Values below the diagonal represent Pearson correlation coefficients (*r*). Values above the diagonal are *p*-values (uncorrected). Values in the last four columns display descriptive data for all tasks. ^∗^*p* < 0.05. ^†^Partial correlation additionally controlling for accuracy on both tasks. ^∧^Reported reliability based on a split-half procedure (McGrew et al., 2007).*

### Children’s Performance on the SNA Tasks

#### WTN Task

Of the total sample, five children were excluded from analyses of the WTN task for failing to complete all four blocks (see **Table 2** for descriptive data). In all analyses of this task, data from the four blocks were combined. Two children were excluded from these analyses due to poor accuracy (>2.5 *SD*s ± *M*), where accuracy was calculated as the absolute distance between the original location of the number and the child’s final placement of the number. The remaining children (*n* = 58) had a mean accuracy of 63.02 pixels [16.67 mm; *SD* = 24.42 pixels (6.43 mm)].

To test for SNAs on this task, the variable of interest was children’s bias along the horizontal axis^3^. For each trial, we calculated the difference between the x-coordinate of children’s final placement and the x-coordinate of the number’s original location, such that a negative value represented a leftward placement in comparison to the original location, and a positive value represented a rightward placement. For each participant, we then calculated the mean bias for each number and calculated a slope by regressing these values onto their corresponding numerical value. Thus, in this task, a positive slope represents the canonical left-to-right mental number line, as a positive slope denotes a shift from leftward to rightward placement, relative to the number’s original position. In other words, just as slopes in the classic SNARC task reflect the extent to which numerical magnitude explains the difference in RTs between left and right hands (Dehaene et al., 1993), slopes in the WTN task reflect the extent to which numerical magnitude explains deviation in the placement of numbers in comparison to the original location.

**FIGURE 1:**
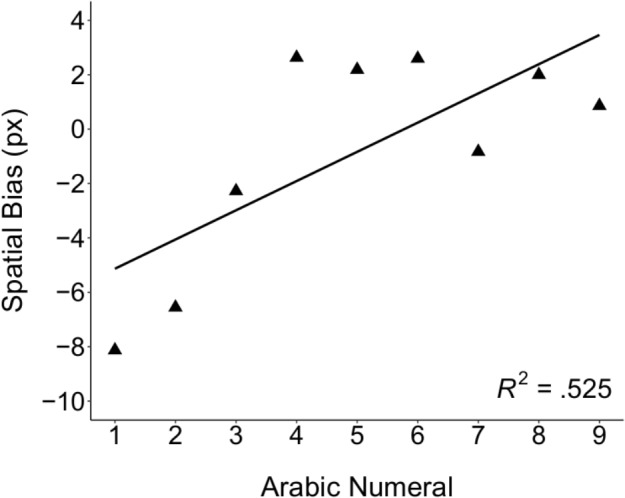
SNA on the WTN task. The mean horizontal bias, in pixels, for each number was regressed on the corresponding numerical value. Numerical value was a significant predictor of mean bias for each number, *t*(8) = 2.78, *p* < 0.05. This result provides evidence for an SNA in children, consistent with a left-to-right oriented mental number line.

Consistent with a left-to-right oriented mental number line that applies to symbolic number, children’s slopes were significantly greater than zero, *t*(57) = 2.02, *p* = 0.048, *d* = 0.265 (see **Figure 1**), and the majority of children (64%) exhibited a positive slope (binomial test, *p* < 0.05). Children’s slopes were not significantly correlated with age, *r*(56) = -0.190, *p* = 0.152, suggesting no relation between symbolic SNAs and age, in a sample of 5- to 7-year-old children. Moreover, children’s slopes were not significantly correlated with overall accuracy, *r*(56) = 0.134, *p* = 0.316, suggesting that children’s SNAs on this task did not vary as a function of their ability to remember the original location of the number.

#### Magnitude Comparison Task

Of the total sample, all children were included in the analyses of the more condition; one child was excluded from the analyses of the less condition due to experimenter error. Children’s performance (proportion correct) was above the chance level of 0.50 in both conditions (more condition: *M* = 0.708, *SD* = 0.147, *t*[64] = 11.41, *p* < 0.001, *d* = 1.42; less condition: *M* = 0.704, *SD* = 0.119, *t*[63] = 13.72, *p* < 0.001, *d* = 1.71), with no significant difference between the two conditions, *t*(63) = 0.008, *p*> 0.99. Consequently, all further analyses utilizing this task were conducted using composite scores of the two conditions.

As a measure of SNAs on this task, we calculated a total congruency score for each child (see **Table 2**). Congruency scores were calculated as the difference between correct congruent and incongruent trials such that a positive congruency score represented a rightward oriented SNA effect. Children exhibited congruency scores (*M* = 0.594, *SD* = 2.23) that were significantly greater than zero, *t*(63) = 2.13, *p* = 0.037, *d* = 0.266, suggesting a left-to-right mental number line that applies to non-symbolic displays of number. Although not a significant majority of children (binomial test, *p* = 0.191), more than half of them displayed positive congruency scores (36 of 64; 56%). Children’s congruency scores were not significantly correlated with age, *r*(62) = -0.210, *p* = 0.095, suggesting no relation between non-symbolic SNAs and age in a sample of 5- to 7-year-old children. Children’s congruency scores were significantly negatively correlated with overall accuracy, *r*(62) = -0.250, *p* = 0.047, but this relation was no longer significant after controlling for age, *r*_p_(61) = -0.191, *p* = 0.134, suggesting no specific relation between congruency scores and accuracy beyond that accounted for by age.

#### Relations Between SNA Tasks

**FIGURE 2:**
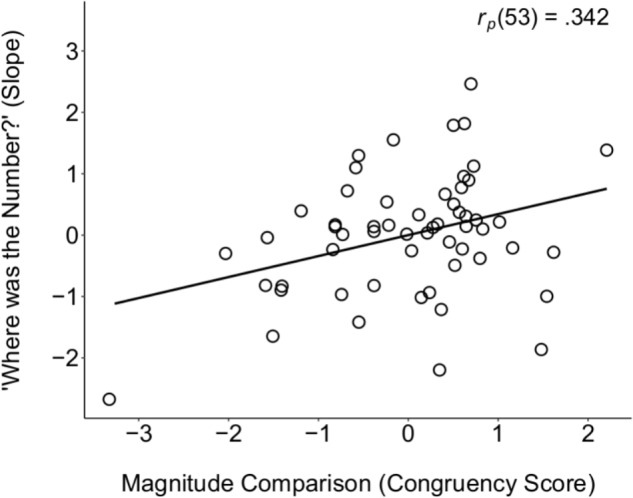
Partial correlation scatter plot between the two SNA tasks (Magnitude Comparison and WTN), when controlling for overall accuracy on both tasks, providing evidence for their convergent validity. No data points qualified as bivariate outliers using the criterion of 2.5^∗^*SD* from the mean.

To test for a potential relation between the two SNA tasks (WTN and magnitude comparison), we conducted correlation analyses between children’s slopes on the WTN task and their congruency scores on the magnitude comparison task. When controlling for accuracy on the two tasks to account for differences in task demands, there was a significant correlation between children’s performance on the two SNA tasks, *r*_p_(53) = 0.342, *p* = 0.011 (see **Figure 2**), demonstrating convergent validity for these SNAs, and suggesting a left-to-right mental number line that is robust to the type of stimuli (symbolic and non-symbolic number). The relation between these two tasks held when additionally controlling for age, *r*_p_(52) = 0.339, *p* = 0.012, suggesting further that the left-to-right mental number line is stable within the age range tested (5 to 7 years). Moreover, in addition to age, this relation held when further controlling for general cognitive abilities – namely, verbal proficiency (WJ–Picture Vocabulary), working memory (WJ–Auditory Working Memory), and short-term memory (K-ABC Spatial Memory), *r*_p_(49) = 0.302, *p* = 0.034 (analyses conducted on raw scores of each task, discussed further below).

### Children’s Performance on the Math Tasks

#### ACA Task

Nine children were excluded from the analyses of the ACA task for failing to complete one or both conditions of this task (see **Table 2** for descriptive data). In the remaining sample (*n* = 56), performance (proportion correct) was significantly above the chance level of 0.50 (*M* = 0.659, *SD* = 0.114), *t*(55) = 10.50, *p* < 0.001, *d* = 1.40. A repeated measures analysis of variance (ANOVA) with operation (addition and subtraction) and ratio (4:5, 4:6, and 4:7) as the within-subjects factors revealed a marginal effect of operation, *F*(1, 55) = 3.81, *p* = 0.056, η*_p_*^2^ = 0.065, such that children performed somewhat better on addition than subtraction trials (Barth et al., 2008). There was also a significant effect of ratio, *F*(2, 110) = 7.11, *p* = 0.001, η*_p_*^2^ = 0.115, and a linear contrast analysis revealed that performance improved as ratio decreased (e.g., better performance for a ratio of 4:7 than 4:5), *F*(1, 55) = 11.64, *p* = 0.001, η*_p_*^2^ = 0.175, as would be expected if the computations were performed approximately (Lipton and Spelke, 2004; Halberda and Feigenson, 2008). There was no interaction between operation and ratio, *p* > 0.94.

#### ASA Task

Thirteen children were excluded from the analyses of the ASA task for failing to complete one or both conditions of this task (see **Table 2**). In the remaining sample (*n* = 53), performance (proportion correct) was significantly above the chance level of 0.50 (*M* = 0.755, *SD* = 0.148), *t*(52) = 12.56, *p* < 0.001, *d* = 1.73. A repeated measures ANOVA with operation (addition and subtraction) and ratio (4:5, 4:6, and 4:7) as within-subjects factors revealed a significant effect of operation, *F*(1, 52) = 4.66, *p* = 0.035, η*_p_*^2^ = 0.082, such that children were more accurate on addition than subtraction trials. There was a marginally significant effect of ratio, *F*(2, 104) = 3.02, *p* = 0.053, The Developing Mental Number Line: Does Its Directionality Relate to 5- to 7-Year-Old Children’s Mathematical Abilities? = 0.055, and a linear contrast analysis revealed a statistically significant linear trend, *F*(1, 55) = 4.45, *p* = 0.040, η*_p_*^2^ = 0.079, such that performance improved as ratio decreased (e.g., better performance for a ratio of 4:7 than 4:5), as expected. There was no interaction between operation and ratio, *p* > 0.13.

#### Exact Symbolic Arithmetic Task

Although the WJ–Calculation subtest allows for computing standardized scores, we instead utilized raw scores in our analyzes, as in other studies (Bugden and Ansari, 2011; Lourenco and Bonny, 2017). The use of raw scores allowed for the inclusion of all children in the subsequent correlation analyses because standardized scores could not be calculated for several children who received scores of zero (*n* = 9). Raw scores on WJ–Calculation ranged from 0 to 14 (*M* = 6.71, *SD* = 4.21). As indicated above, these scores were normally distributed.

#### Analyses of the Relations Between Math Tasks

To our knowledge, previous studies have not examined the relations between the math tasks used in the present study. To assess the potential relations between these tasks, we first conducted a series of partial correlations, controlling for age (see **Table 2** for correlations). Despite acceptable reliability for each measure (*r*s > 0.52), we did not observe significant correlations among the math measures, with the exception of one marginal trend in the relation between tasks that shared a common symbolic format, ASA and WJ–Calculation, *r*_p_(50) = 0.267, *p* = 0.055, such that children who performed better on the ASA task also tended to perform better on WJ–Calculation. These findings are consistent with the literature on math abilities in adults in which dissociations between abilities within the math domain have been reported (e.g., Rosenberg-Lee et al., 2011; Lourenco et al., 2012). Likewise, other work has shown that math abilities are, to some extent, dissociable at younger ages (Fuchs et al., 2010; LeFevre et al., 2010; Cho et al., 2011). Specifically, these dissociations may reflect differences in calculation type (approximate vs. exact), numerical format (non-symbolic vs. symbolic), modality (uni-modal vs. cross-modal), and/or presentation format (simultaneous vs. sequential). Although it is possible that the lack of significant relations observed in the present study could reflect attenuation due to task reliabilities, all reliabilities were in the acceptable range. Therefore, these findings likely reflect early developmental dissociations across different math tasks.

#### Control Tasks

Given that raw scores were used for the WJ–Calculation task, we likewise used raw scores for all of the control tasks. Scores on WJ–Picture Vocabulary, our measure of verbal proficiency, ranged from 14 to 27, with a mean of 20.55 (*SD* = 3.05). Scores on WJ–Auditory Working Memory, our measure of verbal working memory, ranged from 0 to 25, with a mean of 13.32 (*SD* = 6.09). Scores on K-ABC Spatial Memory, our measure of spatial short-term memory, ranged from 5 to 16, with a mean of 11.11 (*SD* = 3.10). As indicated above, these scores were normally distributed.

### Is There a Relation Between Children’s SNAs and Math Performance?

We conducted correlation analyses between children’s performance on the two SNA tasks and each math task, controlling for age, to address the main question motivating the present work. When the WTN task served as the SNA measure, we found no significant correlations between children’s slopes on the WTN task and their accuracy on any math task (see **Table 2**). In particular, there were no relations between children’s performance on WTN and ACA tasks. Furthermore, there were no relations between children’s performance on the WTN task and either symbolic arithmetic task (i.e., ASA and WJ–Calculation).

**FIGURE 3:**
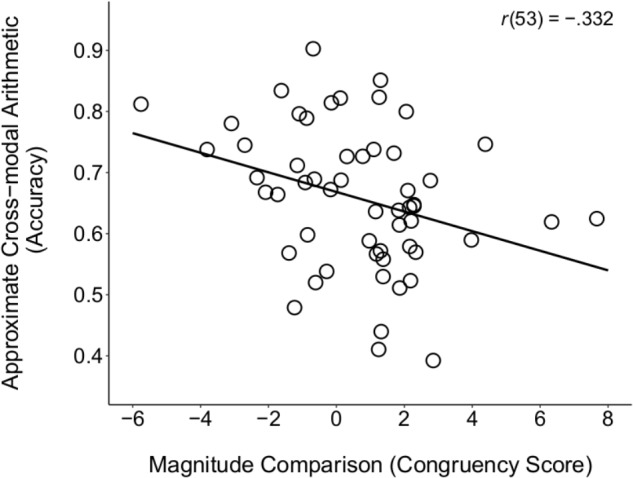
Scatterplot depicting the zero-order correlation between children’s congruency scores on the Magnitude Comparison task and accuracy on the ACA task. Children with stronger SNAs, as indexed by the Magnitude Comparison task, displayed poorer performance on non-symbolic arithmetic involving stimuli from different modalities (vision and audition). No data points qualified as bivariate outliers using the criterion of 2.5^∗^*SD* from the mean.

When using congruency scores on the magnitude comparison task as the measure of SNAs, we found a significant correlation with performance on the ACA task, *r*_p_(52) = -0.313, *p* = 0.021 (see **Figure 3**; *p*s > 0.15 for all other correlations between the magnitude comparison task and math ability, see **Table 2**). This negative correlation suggests that a stronger SNA was related to poorer understanding of cross-modal number representations that required arithmetic operations. Moreover, this effect held when additionally controlling for children’s verbal proficiency (WJ–Picture Vocabulary), working memory (WJ–Auditory Working Memory), and short-term memory (K-ABC Spatial Memory), *r*_p_(49) = -0.314, *p* = 0.025, suggesting a robust relation not due to these particular cognitive abilities. But could poor numerical precision (Halberda and Feigenson, 2008) and, thus, difficulty distinguishing smaller and larger numerical arrays, account for the significant correlation? We addressed this possibility directly by controlling for children’s accuracy on the magnitude comparison task, in addition to age and general cognitive ability. The relation between children’s SNAs, as indexed by congruency on the magnitude comparison task, and ACA performance, remained statistically significant, *r*_p_(48) = -0.290, *p* = 0.041. Thus, although there was only one significant correlation between children’s SNAs and their math ability in the present study, this effect held when controlling for other cognitive abilities and when addressing an alternative account based on poor numerical precision. This finding suggests that there is a negative relation between the directional mental number line, as assessed by the magnitude comparison task, and the understanding of abstract (i.e., modality-independent) numerosity. We discuss this negative relation in the Section “General Discussion.”

### General Discussion

The primary goal of the present study was to examine the potential relations between SNAs and emerging mathematical competence in childhood. Although much interest has concerned the spatial nature of number representations, we know little about the links between these representations and mathematical development. As discussed in the Section “Introduction,” existing research on this topic has been mixed (e.g., Hoffman et al., 2013; Gibson and Maurer, 2016). Here we adopted two measures of SNAs and multiple measures of math competence in an effort to shed light on the important question of whether the directionality of the mental number line may offer functional significance in the domain of mathematics, particularly at an age when quantitative reasoning is undergoing development.

Our two measures of SNAs revealed left-to-right orientation of number representations in 5- to 7-year-olds. We showed this effect with a non-symbolic magnitude comparison task, which has been used in previous work with children (Patro and Haman, 2012), as well as the novel, symbolic WTN task, only used previously with adults (Aulet et al., 2017). Importantly, not only did we find evidence of left-to-right orientation of number on both tasks, but we also found a correlation between performance on these tasks, even when controlling for accuracy, age, and general cognitive abilities, thereby providing convergent evidence of a mental number line early in development. Even in adults, it is rare to assess construct validity of SNAs (for exceptions, see Cheung et al., 2015; Georges et al., 2017a). Here, we show that SNAs can be captured with different tasks in children, such that individual differences in the strength of these SNAs were common across tasks.

We also examined the relation between each SNA task and children’s performance on a variety of measures designed to tap basic mathematical competence. We observed no significant correlations between slopes on the WTN task and children’s performance on the math tasks, suggesting no relation between SNAs and early math abilities. However, could other factors account for the lack of correlations between the WTN task and math performance? One possibility is that slopes on this task underestimated the directionality of the mental number line for children with more compressive mental number lines. Visual inspection of **Figure 1** certainly suggests a non-linear relation between numerical value and spatial bias, which may reflect compressive representations of number on this task. As the goal of the present study was to assess the relation between directionality and math ability, we did not systematically investigate spatial scaling of the mental number line on the WTN task. Nonetheless, although we cannot rule out this possibility directly, we think it is unlikely that slopes were systematically underestimated given that the majority of children’s responses were consistent with a rightward-oriented mental number line. Moreover, we observed a significant positive correlation between children’s slopes on the WTN task and congruency scores on the magnitude comparison task, which would not be expected if the underestimation of slopes on the WTN task resulted in a failure to capture individually differences in the directionality of children’s mental number lines. Thus, although it is possible that individual differences in spatial scaling may have impacted the precision of the estimates of directionality on the WTN task, this alone likely cannot account for the non-existent relations between WTN slopes and mathematical ability.

By contrast, there was a relation between children’s congruency scores on the magnitude comparison task and their performance on the ACA task, but this relation was negative, which we did not predict for children between 5 and 7 years of age. In particular, we found that 5- to 7-year-olds with stronger SNAs (i.e., larger congruency scores) performed worse on the ACA task, even after controlling for age, general cognitive abilities, such as working memory, and accuracy on the magnitude comparison task itself. As discussed in the Section “Introduction,” although previous studies in children have typically reported a positive relation between SNA strength and math ability (Bachot et al., 2005; Georges et al., 2017b), our findings mirror previous studies in adults that have also reported a negative relation between SNA strength and mathematical ability (Hoffmann et al., 2014a; Cipora et al., 2016). At minimum, the data observed in the present study would seem to suggest that children with a more robust left-to-right mental number line perform at a level below their peers in mathematics. This negative relation could be interpreted as suggesting that a directional mental number line hinders, rather than facilities, mathematical development. Such an interpretation, however, is at odds with a large literature on the role of analogy (Gentner et al., 2001; Siegler, 2016), metaphor (Núñez and Lakoff, 2005), and embodiment (Barsalou, 2008) in the acquisition and understanding of abstract concepts.

One possible explanation for the negative relation between children’s math performance and the magnitude comparison task, but not the WTN task, is that this relation may reflect additional task-specific demands. In particular, the congruency effect on the magnitude comparison task, which was used to assess the strength of children’s SNAs, might reflect inhibitory control required by this specific task and potentially associated with mathematical competence (Fuhs and McNeil, 2013; Cragg and Gilmore, 2014; Hohol et al., 2017). Successful performance on the magnitude comparison task required an assessment of which array was smaller or larger in numerosity, regardless of the spatial position of the arrays. On the incongruent trials, this might involve inhibition of the mental number line, since, on these trials, the correct array was in the spatially incongruent position. As a consequence, inhibition of the mental number line would actually result in greater accuracy on these incongruent trials. Thus, smaller congruency scores could indicate a weak SNA or could, instead, indicate an inability to inhibit an SNA when it conflicted with the goal of the task. By contrast, the WTN task required no such inhibitory demands and, as discussed earlier, this task was not correlated with any of the math measures given to children.

The ACA task, like the magnitude comparison task, displayed arrays on the left and right sides of the screen. Could this common spatial layout therefore explain the negative relation between congruency scores on the magnitude comparison task and accuracy on the ACA task? Although we cannot rule out this possibility directly, we would suggest that it is unlikely because the ASA task also shared this layout, and there was no relation between congruency scores on the magnitude comparisons task and accuracy on the ASA task. Thus, the common layout between tasks would appear insufficient to explain the negative relation observed between SNAs and math ability in the present study. What, then, might account for this finding? Successful performance on the ACA task might also depend on inhibitory control, similar to the suggestion by Fuhs et al. (2016) that a relation between executive function and math achievement arises from the ability to accurately represent the value of a numerical set as opposed to the individual items within a set. In the ACA task, numerosities were presented across different modalities (vision and audition) and presentation formats (simultaneous vs. sequential). On this task, in contrast to the ASA task, children had to abstract numerical value over quite disparate stimuli. The differences in modality and presentation format might have increased the salience of the individual items, requiring more inhibition to delay responses until the value of the full set could be assessed. If inhibitory control were necessary to assess the set as a whole, then individual differences in inhibitory control would influence performance on the ACA task. As a consequence, poor inhibitory control could lead to both larger congruency scores on the magnitude comparison task and worse performance on the ACA task (for a similar finding, see Hoffmann et al., 2014b).

Given the alternative explanation just described, and the lack of significant correlations involving the WTN task (our other measure of SNAs), our findings do not provide strong support for a relation between a directional mental number line and math competence in 5- to 7-year-old children. Importantly, the inhibitory control account of the negative relation between SNAs, as indexed by the magnitude comparison task, and accuracy on the ACA task, does not suggest that the mental number line itself is negatively related to math ability. Rather, tasks such as the magnitude comparison task may require inhibition of the mental number line for optimal performance and other tasks may depend on inhibitory control more generally for performing numerical comparison and/or arithmetic computation across numerical format (Fuhs et al., 2016). Thus, it remains possible that, in the absence of such inhibitory control demands, there may exist a positive relation between SNAs involving non-symbolic numerosities and math ability.

As we outlined in the Section “Introduction,” Hoffman et al. (2013) found a positive correlation between SNAs and math ability. Interestingly, they used a magnitude comparison task, as in the present study, but with numerals. This study, however, did not find an overall effect of SNAs for the group of children tested, nor were there controls for general cognitive functioning, which could account for a correlation between performance on their magnitude comparison task and numerical proficiency. As in the present study, it would be especially important to determine the extent to which inhibitory control might account for the correlation in Hoffman et al. (2013). Other published work has found no significant effects between the strength of children’s SNAs and performance on a math test (Gibson and Maurer, 2016). This study also did not include measures of general cognitive functioning, such that it is unclear to what extent inhibitory control or other variables could have accounted for the results. It is also possible that age may play a role in determining the relation between SNAs and math ability. An important difference between these studies is that the children in the Hoffman et al. (2013) study were younger than those tested here and in the study of Gibson and Maurer (2016). Thus, a positive SNA-math link could exist earlier in development, such that a mental number line might prove beneficial to mathematical reasoning, but this link is only present during the earliest stages of acquisition when young children are first learning quantitative concepts and operations such as those tested in the present study.

An important consideration for this research program going forward is whether an individual differences approach, adopted here and in other studies, is well suited for assessing whether a mental number line benefits math development. In particular, we took the approach that if the directionality of the mental number line were relevant for math development, then one should observe a correlation between the strength of one’s SNA and performance on one or more of the math tasks administered to children. However, it is possible that some minimal amount of left-to-right organization in one’s number representations is sufficient for supporting learning of abstract number concepts or performing arithmetic computations. If minimal organization were sufficient, then relations between tasks assessing SNAs and children’s math performance would not be observed.

Another important consideration that follows from the current and existing research is that other components of the mental number line, besides directionality, may be related to mathematical competence (for review, see Cipora et al., 2015). In the Section “Introduction,” we hypothesized that the spatial grounding provided by a mental number line might facilitate understanding of number as an abstract concept and, thus, a stronger left-to-right orientation of number would provide support for math tasks, such as cross-modal arithmetic, that rely on this abstract understanding (Lakoff and Núñez, 2000; Barsalou, 2008). We also hypothesized that directionality could be beneficial for performing arithmetic. Effects of operational momentum in which individuals associate larger outcomes with addition and smaller outcomes with subtraction (McCrink et al., 2007; Knops et al., 2009) are consistent with shifts of attention along a mental number line during these arithmetic operations. The ability to dynamically shift one’s attention in relation to this spatial representation may be comparable to other visuospatial processes such as mental rotation that have been shown to relate to mathematical reasoning (Thompson et al., 2013; Cheng and Mix, 2014). The magnitude comparison and WTN tasks, however, were designed to capture the extent of left-to-right orientation, not the dynamic quality of the mental number line, or of attentional processes that may be applied to it, which, ultimately, may be more predictive of math development.

Another critical feature of the mental number line is the spatial scaling of numerical intervals. Rather than direction (e.g., left-to-right), we can ask whether the scaling is best characterized by a linear or logarithmic mapping of number to spatial extent. Most commonly, these mappings are measured by a number line estimation task where participants designate the position of a numerical value on a physical line anchored by two numbers (Siegler and Opfer, 2003). In the case of a linear representation, a change in numerical distance corresponds to an equivalent change in spatial distance. That is, across the entire range of the number line, numerical values are represented with consistent spatial intervals when the representation is linear. Conversely, for compressive representations, the spatial distance between two small numbers is judged as larger than that between two larger numbers of equivalent numerical difference (e.g., children designate the numbers 5 and 15 as farther apart than 75 and 85; but, see Barth et al., 2011; Cohen and Quinlan, 2017). Not only has the linearity of one’s number line been shown to correlate positively with math proficiency, as measured by a variety of math measures, but causal evidence has also been put forth, in which children who receive training to increase the linearity of their numerical representations subsequently show better math scores than those receiving non-numerical (control) training (for meta-analysis, see Schneider et al., 2018). Thus, although there may be little evidence for a relation between the directionality of the mental number line and mathematical competence, there is accumulating support for the importance of a linear mental number line in math development.

## Conclusion

In conclusion, our results do not provide strong support for a relation between a directional mental number line and mathematical ability in 5- to 7-year-old children. Contrary to our initial prediction, the sole significant SNA-math relation was negative, such that a stronger SNA was associated with worse, not better, performance on a measure of early mathematical competence. Though we have suggested that this link is likely due to individual differences in inhibitory control, consistent with previous research (Hoffmann et al., 2014b), we acknowledge the speculative nature of this claim given that the present study did not include a direct measure of inhibition. Thus, we urge future research on this topic to consider the potential influence of inhibitory control on different measures. Moreover, additional research is necessary to determine whether children younger than those tested here are more likely to benefit from a left-to-right oriented mental number line, and further, whether different facets of the mental number line, such as directionality and scale, contribute differentially to mathematical development. We also encourage researchers to consider experimental designs beyond an individual differences approach to shed light on these questions.

## Ethics Statement

This study was carried out in accordance with the guidelines of the Institutional Review Board (IRB) at Emory University under IRB protocol #003571. The protocol was approved by the IRB at Emory University. The parent or guardian of all participants gave written informed consent in accordance with the Declaration of Helsinki.

## Author Contributions

LA and SL conceived and designed the experiments, and wrote the paper. LA performed the experiments and analyzed the data.

## Conflict of Interest Statement

The authors declare that the research was conducted in the absence of any commercial or financial relationships that could be construed as a potential conflict of interest.

## Footnotes

1 In the present study, we define the mental number line as a representation of number in which numerical value is associated with a spatial location. The spatial properties of the mental number line include both directionality (i.e., the specific directional orientation of the line) and scaling (i.e., the spatial intervals between numerical values). We define spatial-numerical associations (SNAs) as the behavioral manifestations of these properties, though the current work concerned directionality in particular. To this end, we describe SNAs here as behavioral manifestations of the directionality of the mental number line.

2 Despite the left-right presentation of displays of the ACA and ASA tasks, an assessment of SNAs on these tasks is not straightforward and, thus, was not implemented. The reason is that the presentation of displays may have elicited additional biases, such as choosing the display that was added to on the addition trials (left display) and choosing the display that was not subtracted from on the subtraction trials (right display). Indeed, some children exclusively chose the left or right display in these tasks (see Results).

3 Analysis of children’s bias along the vertical axis yielded no evidence of a significant SNA, *p* > 0.70. Previous research suggests SNAs along the vertical axis may be less robust than those along the horizontal axis (Holmes and Lourenco, 2012). However, it is also possible that the rectangular space used here constrained bias in the vertical dimension.
